# Associations between structure and function are different in healthy and glaucomatous eyes

**DOI:** 10.1371/journal.pone.0196814

**Published:** 2018-05-03

**Authors:** Fang-I Chu, Iván Marín-Franch, Koosha Ramezani, Lyne Racette

**Affiliations:** 1 Eugene and Marilyn Glick Eye Institute, Indiana University, Indianapolis, Indiana, United States of America; 2 Department of Ophthalmology and Visual Sciences, University of Alabama at Birmingham School of Medicine, Birmingham, Alabama, United States of America; Bascom Palmer Eye Institute, UNITED STATES

## Abstract

**Purpose:**

To assess if there are differences in the structure-function associations between healthy and glaucomatous eyes.

**Methods:**

Structure-function associations were assessed in healthy and glaucomatous eyes in three datasets, globally and in the six sectors of the optic nerve head. Structural parameters included rim area (RA) and retinal nerve fiber layer thickness (RNFLT). Functional parameters included unweighted mean of sensitivity thresholds (MS) and unweighted mean of total deviation values (MD), assessed with standard automated perimetry, short-wavelength automated perimetry, frequency-doubling technology perimetry, or contrast sensitivity perimetry. All structural and functional parameters were expressed as percent of mean normal. SF associations were assessed with correlation analyses (Pearson, Spearman and Kendall). We also assessed the SF associations with linear regression analyses: the generalized estimating equation (GEE) was used to adjust for inter-eye correlations and ordinary least squares (OLS) linear models were used when these adjustments were not necessary. We applied Bonferroni corrections to adjust for the impact of multiple comparisons.

**Results:**

Overall, none of the Pearson correlations tested in healthy eyes were significant (correlations ranged from -0.17 to 0.37), whereas 77% of the correlations tested in glaucomatous eyes were significant (correlations ranged from 0.01 to 0.79). Similarly, none of the slopes obtained with GEE and OLS were significant in healthy eyes (slopes ranged from -0.30 to 0.87), whereas 82% of the slopes obtained in glaucomatous eyes were significant (slopes ranged from 0.02 to 1.38).

**Conclusions:**

Significant associations between structure and function were consistently observed in glaucomatous eyes, but not in healthy eyes. These differences in association should be considered in the design of structure-function models for progression.

## Introduction

Primary open-angle glaucoma (POAG) is a chronic optic neuropathy characterized by the progressive degeneration of the retinal ganglion cells and their axons.[1] Ganglion cell degeneration results in morphological changes to the optic nerve head and retinal nerve fiber layer, and also in the loss of visual function. Some degree of association is therefore expected between structural damage and loss of visual function in glaucomatous eyes.[2] Both histological[3–7] and clinical[8–13] studies have shown the existence of correlations between structure and function in glaucomatous eyes. In healthy eyes, however, the relationship between structure and function remains unclear.

Models that characterize the structure-function (SF) relationship in glaucoma make different assumptions about whether SF associations are different or not in healthy and glaucomatous eyes. Some models assume a linear (or monotonic) association between structure and function when data from both healthy and glaucomatous eyes are pooled together.[14–16] Other models assume that there is no association between structure and function in healthy eyes.[17–19] These assumptions have important implications because they can lead to different conclusions about the form (linear or curvilinear) and the strength of the SF relationship. Consequently, the assumptions may impact models that use structural and functional data jointly for the detection of glaucoma and its progression.[14, 18]

While the observed SF relationship is influenced by several factors,[20] the inconsistent results observed between studies[21] regarding the relationship between structural and functional parameters may be due in part to the assumptions made about the associations in healthy eyes. The aim of this study was to determine whether differences exist in the SF associations between healthy and glaucomatous eyes.

## Methods

This secondary data analysis adheres to the tenets of the Declaration of Helsinki. Institutional review board (IRB) approval to perform this study was initially obtained from Indiana University, and then from the University of Alabama at Birmingham following the relocation of the laboratory of the corresponding author. IRB approval was also obtained at each of the institutions at which the original datasets were collected. The SF associations in healthy and glaucomatous eyes were assessed using three different datasets to ensure that our findings are not due to the intricacies of one specific dataset. We used the Diagnostic Innovations in Glaucoma Study and the African Descent and Glaucoma Evaluation Study (DIGS-ADAGES) dataset[22] and a dataset from the State University of New York and Indiana University (SUNY-IU)[23] that each had healthy and glaucomatous eyes and a dataset from the University of Iowa with only healthy eyes.[19] Written informed consent was obtained from each participant. All studies were approved by the appropriate Institutional Review Boards, adhered to the tenets of the declaration of Helsinki for research involving human subjects, and were performed in conformity with the Health Insurance Portability and Accountability Act (HIPAA).

### Datasets

#### The DIGS-ADAGES dataset[22]

These two, ongoing, longitudinal multi-center studies were prospectively designed to assess structure and function in glaucoma. They were conducted at the University of California San Diego (UCSD), the New York Eye and Ear Infirmary and the University of Alabama. Participants with healthy and glaucomatous eyes were selected based on the definition of healthy and glaucomatous eyes outlined in Table 1 of Sample et al.[22] In brief, healthy eyes were those in the normal diagnostic category, while glaucomatous eyes were those that had both abnormal visual fields test result and glaucomatous optic neuropathy.

**Table 1:** Number of observations and power for analysis for the critical value of 0.2 of Pearson correlation.

	Healthy eyes		Glaucomatous eyes	
	Subjects (eyes)	Power	Subjects (eyes)	Power
DIGS/ADAGES				
RA vs SAP MS and MD	694 (1212)	1.00	271 (362)	0.91
RA vs SWAP MS and MD	472 (802)	0.99	247 (328)	0.89
RA vs FDP MS	685 (1207)	1.00	266 (356)	0.91
RNFLT vs SAP MS and MD	204 (336)	0.82	180 (238)	0.77
RNFLT vs FDP MS	181 (295)	0.77	151 (200)	0.70
IOWA				
RNFLT vs SAP MD	76 (76)	0.41		
SUNY-IU				
RNFLT vs SAP, CSP, FDP MS	62 (62)	0.35	51 (51)	0.29

In this dataset, rim area (RA) was collected using the Heidelberg Retina Tomograph (Heidelberg Engineering, Heidelberg, Germany), and retinal nerve fiber layer thickness (RNFLT) was collected with optical coherence tomography (OCT) using the Stratus OCT (Carl Zeiss Meditec, Dublin, CA). Standard automated perimetry (SAP) (Carl Zeiss Meditec, Dublin, CA), short-wavelength automated perimetry (SWAP) (Carl Zeiss Meditec, Dublin, CA), and frequency-doubling perimetry (FDP) (Carl Zeiss Meditec, Dublin, CA) were also collected. Healthy and glaucomatous eyes from this dataset were included in the present retrospective cross-sectional study if they had at least one pair of SF data taken within a 30-day period. When several SF pairs were available, the first SF pair was selected for all subjects. This minimized the risk of including eyes that may have developed early glaucoma in the healthy eyes group. While this could have increased the risk of including visual fields with learning effects, the protocol of these studies required that all subjects be given practice tests if they were new to perimetry.[24] In addition, we only included visual fields that were determined to have no learning effect by the Visual Field Assessment Center (VisFACT) reading center at UCSD[24]. We also required the presence of at least 50 eyes for each SF pair. When available, eligible data from both eyes were included.

#### The IOWA dataset[19]

The IOWA dataset consisted of subjects with healthy eyes obtained from two sources. The first group, referred to as the long-term repeat group,[19] consisted of 60 healthy eyes from 60 subjects that had 2 visits each. The second group, referred to as the short-term repeat group,[19] consisted of 22 healthy eyes from 22 subjects with 4 or 5 visits each all taken within a 60-day window (two subjects had only 4 visits[19]). RNFLT was measured with OCT3; Stratus fast RNFL circular scan; Carl Zeiss Meditec) and SAP was obtained with the Humphrey Field Analyzer, program 24–2 SITA standard (Carl Zeiss Meditec, Dublin, CA). Learning effects were not directly assessed in the IOWA dataset. We therefore discarded the first visit to minimize the impact of possible learning effects and averaged the remaining visits. While we report the results of the analyses in which the first visit was excluded, it should be noted that all results were similar when we included all visits in the analyses. For the present study, we pooled the data from these two sources and used a sample of 76 healthy eyes from 76 subjects. Data from glaucomatous eyes were not available in this dataset and it therefore could not be used to directly determine whether differences in SF associations between healthy and glaucomatous eyes exist. We nonetheless included this dataset because it allows us to assess the SF associations in healthy eyes and to compare them to those obtained in the other datasets used here.

#### The SUNY-IU dataset[23]

Participants included in this dataset were enrolled in a multicenter longitudinal study at three different university clinics. One center was at the State University of New York (SUNY) and the other two centers were affiliated with Indiana University, one in Indianapolis and one in Bloomington. In brief, 62 participants with healthy eyes and 51 patients with glaucoma were included. Patients with glaucoma had visual field loss and glaucomatous optic neuropathy. RNFLT was measured with Stratus OCT3, Model 3000(Carl Zeiss Meditec, Inc), SAP was obtained with the HFAII, 24–2 SITA standard (Carl Zeiss Meditec, Dublin, CA), FDP was performed with the Humphrey Matrix (Carl Zeiss Meditec, Dublin, CA) using the 24–2 test pattern, and contrast sensitivity perimetry (CSP) testing was performed using custom testing stations based on 21-inch cathode-ray tube displays driven by a visual stimulus generator (ViSaGe; Cambridge Research Systems, Ltd, Rochester, Kent, UK). One eye of each participant was tested on a single visit. As for the DIGS-ADAGES dataset, all subjects had previous experience with perimetry, so no learning effect was expected.[23]

### Structural and functional tests and parameters

We assessed the SF associations in several combinations of structural and functional parameters to characterize the SF relationship as comprehensively as possible. All parameters included in this study are the ones available in the DIGS-ADAGES, IOWA and SUNY-IU dataset. The structural parameters included RA and RNFLT. Functional parameters included unweighted mean of the 52 sensitivity thresholds (MS) of the 24−2 test (the two locations above and below the blind spot were excluded) and the unweighted mean of total deviation values (MD). We used this definition of MD[18] instead of the instrument-generated MD because we assessed the SF association globally and in each of six sectors.

These parameters were obtained for SAP, SWAP, FDP, and CSP when available. The detailed description of each of the structural and functional tests and parameters can be found in the papers cited above for each study.[19, 22, 23]

#### Structure-function pairs

In the DIGS-ADAGES dataset, the following eight combinations of parameters for SF pairs were assessed: RA-MS (SAP, SWAP and FDP), RA-MD (SAP and SWAP), RNFLT-MS (SAP and FDP), RNFLT-MD (SAP). The SF associations were assessed globally (G), and in the following sectors: temporal (T), supero-temporal (ST), infero-temporal (IT), nasal (N), supero-nasal (SN), and infero-nasal (IN) based on the Garway-Heath map.[8] In the SUNY-IU dataset, the RNFLT-MD pair was assessed using SAP, FDP and CSP in the ST and IT sectors. Finally, in the IOWA dataset, SF associations were assessed using the RNFLT-MD parameters globally and in the ST and IT sectors.

### Statistical analysis

To assess the associations between structural and functional measurements, all data were expressed in percent of mean normal. For MS, this was done after converting the sensitivities at each location from a logarithmic scale (dB) to a linear scale (1/Lambert).[25] The values were then averaged across locations to obtain the MS value. For MD, we first converted the total deviation (TD) values from the logarithmic units to linear units, and then averaged them across the 52 locations.[18] All values were then expressed as percent of mean normal (the average obtained for healthy eyes for each dataset included in the study).

We conducted two types of analyses: 1) correlation analyses after selecting one eye at random from subjects for which both eyes were included in the study and 2) linear regression analyses with all eyes available to accommodate for the fact that some subjects had both eyes included in the study. All the statistical analyses were made with R.[26]

#### Correlation analyses

For all three datasets, we assessed the SF associations using Pearson, Spearman, and Kendall correlations with a subset of data that met the underlying assumption of independence, that is, all pairs of measurements are independent from each other. For the DIGS-ADAGES dataset in which both eyes of some subjects were included, one eye was selected at random and the contralateral eye was discarded from the analysis. While Pearson correlation assumes a linear association between the data, both Spearman and Kendall correlations assume a monotonic association. The calculations of Spearman correlation are based on rank correlations, whereas those of Kendall correlation are based on concordant versus discordant pairs. For all correlation analyses, the *p*-values were calculated for the significance test of zero correlation. In addition, we computed the statistical power for the Pearson correlation significance test using the alternative hypothesis of correlation of 0.2, a correlation regarded to be small enough for any practical uses and often labeled as “weak” or negligible.[27]

#### Linear regression analyses

In this approach, the functional measure was treated as the response variable and the structural measure as the explanatory variable, as has traditionally been done. The unit effect from the structural on the functional measure indicates how much function changes per one unit of change on structure. In the DIGS-ADAGES dataset, data were available for two eyes for some of the participants. The SF associations were therefore assessed using the generalized estimating equation (GEE), which adjusts for potential correlations between the two eyes of the same participant. GEE also allows to compensate for age effects in MS and MD (even though MD is already age-corrected). The unit effect in GEE has a similar interpretation as in ordinary least square (OLS) regression: change in response per unit change in explanatory variable. The unit effect in GEE estimates the average effect over the population and was assumed with an exchangeable working correlation to account for within-subject correlations. An additional analysis using a linear mixed effect model (LMM) was also performed that, as GEE, accounts for the presence of both eyes in some subjects in the DIGS-ADAGES dataset. For LMM, the unit effect estimates the marginal effect as the fixed effect since the within-subject correlations are accounted through the random effect term in LMM. For the IOWA and the SUNY-IU datasets, we used OLS which was sufficient to analyze the data obtained from one eye per subject. For all linear regression analyses, the *p*-values were calculated for the significance test of zero slope.

We applied Bonferroni corrections to adjust for the impact of multiple comparisons. Thus, significance level was set at 0.05 divided by the respective number of tests performed in each analysis. Normality and linearity of associations were assumed for GEE, LMM, and OLS, as well as for the significance test for Pearson correlation.

## Results

### Correlation analyses

**Fig 1:**
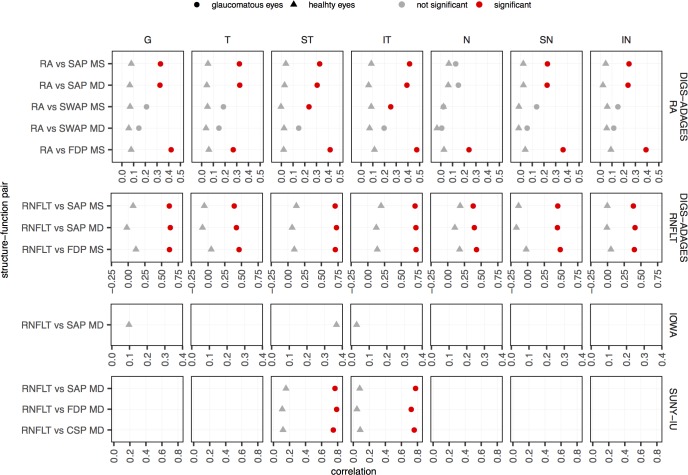
Pearson correlation for each of the structure-function pairs. Results are presented globally and in all sectors for healthy (triangles) and glaucomatous (circles) eyes. Correlations that were found to be significantly different from zero after Bonferroni correction are shown in red. Note that the range of the x-axes are different for the different datasets; we plotted the graph using the range observed in each dataset to highlight the differences between healthy and glaucomatous eyes in each dataset.

Fig 1 summarizes the Pearson correlations for the SF pairs in all sectors in the three datasets. The results for Spearman and Kendall correlations were similar to the ones obtained for Pearson correlation and are presented as supporting information in S1 and S2 Figs, respectively. Table 1 shows the sample size and statistical power of the significance tests of zero correlation computed against the alternative hypothesis of a 0.2 correlation.

Overall, no significant correlations were found for healthy eyes (correlations ranged from -0.17 to 0.37), whereas 48 out of 62 correlations (77%) were significant for glaucomatous eyes (correlations ranged from 0.01 to 0.79). In the DIGS-ADAGES dataset, similar results were observed in glaucomatous eyes when RA and RNFLT were used as the structural test. All correlations were significant in glaucomatous eyes, except when SWAP data was used. For the SUNY-IU dataset, all 6 correlations for glaucomatous eyes and none for healthy eyes were significantly different from zero.

### Linear regression analyses

**Fig 2:**
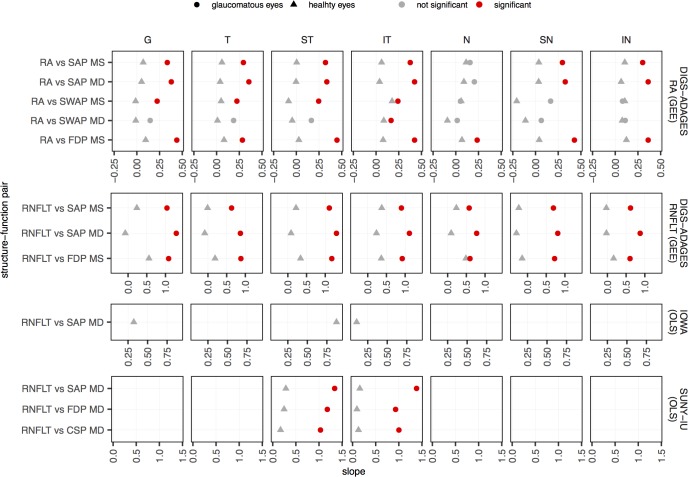
Unit effect (slopes) estimated with GEE or OLS regression of function on structure for each of the structure-function pairs. Results are presented globally and in all sectors for healthy (triangles) and glaucomatous (circles) eyes. Slopes that were found to be significantly different from zero after Bonferroni correction are shown in red. Note that the range of the x-axes are different for the different datasets; we plotted the graph using the range observed in each dataset to highlight the differences between healthy and glaucomatous eyes in each dataset.

Fig 2 summarizes the unit effects estimated with GEE and OLS for function on structure for the SF pairs globally and in all sectors available in the three datasets. Similar results were obtained when these analyses were performed for structure on function (see supporting information in S3 Fig) and for the LMM analysis (see supporting information in S4 Fig).

Overall, no significant slopes were found for healthy eyes (slopes ranged from -0.30 to 0.87), whereas 51 out of 62 correlations (82%) were significant for glaucomatous eyes (correlations ranged from 0.02 to 1.38). Except for the pairs that included SWAP, all slopes were significantly different from zero for glaucomatous eyes. For the SUNY-IU dataset, all 6 OLS slopes for glaucomatous eyes and none for healthy eyes were found significantly different from zero.

## Discussion

In the present study, we consistently found that the associations between structure and function are different in healthy eyes compared to glaucomatous eyes. In healthy eyes, no associations were found between structure and function, while significant associations were found in glaucomatous eyes for several of the comparisons we performed. This robust finding was observed globally and in each of the sectors we assessed, in each of the analyses we performed, as well as across all datasets.

The different associations between structure and function in healthy and glaucomatous eyes need to be taken into account when studying the SF relationship in glaucoma. These differences also have implications for the development of models that use both structural and functional parameters to monitor glaucoma progression. Our results support the assumptions made by the Hood and Kardon model,[18, 19] that there is negligible or no correlation between structural and functional measurements in healthy eyes. Our results, however, are in disagreement with previous results[15] and the assumptions made in other models, such as the Bayesian linear regression[14] and sANSWERS[28] that the associations between structure and function are linear with the same slope for healthy and glaucomatous eyes. Pooling healthy and glaucomatous eyes together leads to models that are not representative of either group. For instance, if we fit a line to the observed structure and function data (with e.g. OLS, GEE, or LMM), the slope will underestimate the results for glaucomatous eyes while overestimating the results for healthy eyes. The biases generated in both healthy and glaucomatous eyes that occur when pooling the data from both groups are bound to reduce the sensitivity of glaucomatous progression of models that use structural and functional information. In this study, we only applied statistical methods that describe linear or monotonic associations because non-monotonic associations (e.g. sinusoidal or other where, for example, RA passes from increasing to decreasing with MS) are biologically implausible.

**Fig 3:**
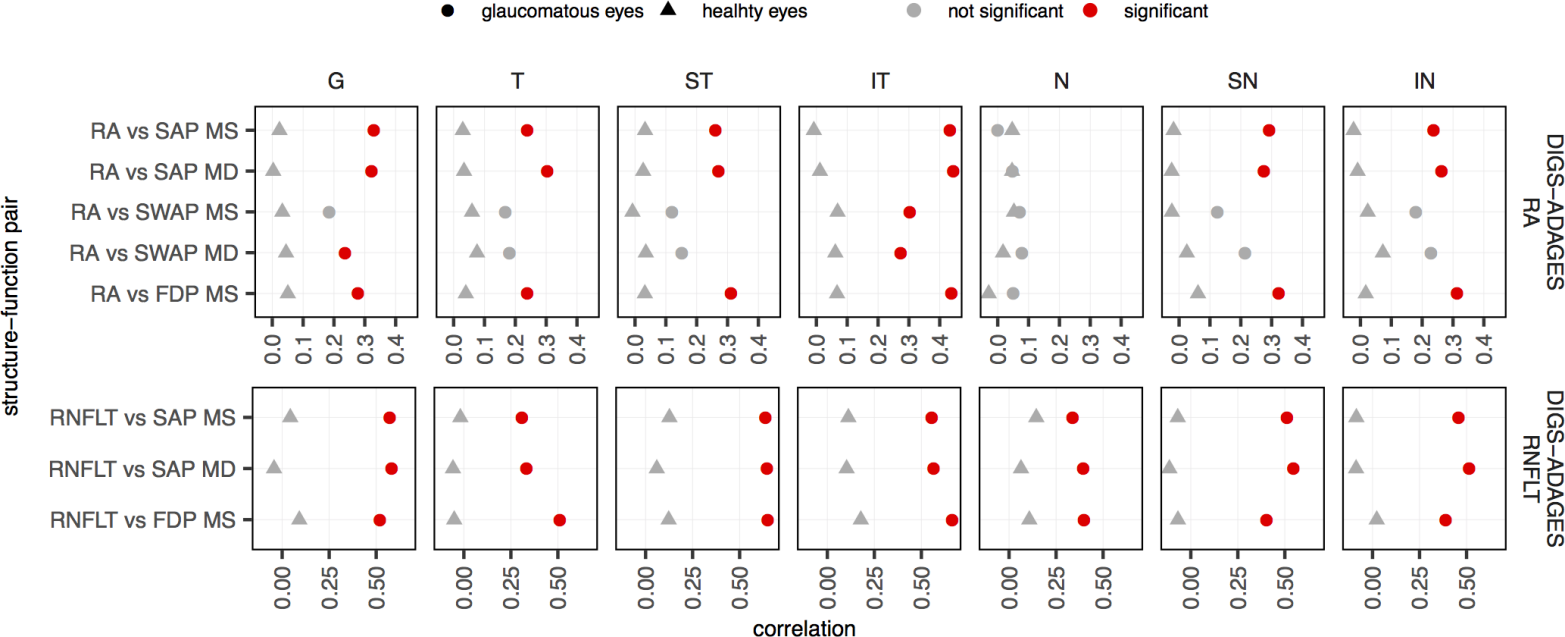
Pearson correlation for each of the structure-function pairs for DIGS-ADAGES dataset after restricting the range for glaucomatous eyes. Results are presented globally and in all sectors for healthy (triangles) and glaucomatous (circles) eyes. Correlations that were found to be significantly different from zero after Bonferroni correction are shown in red. Note that the range of the x-axes are different for the different datasets; we plotted the graph using the range observed in each dataset to highlight the differences between healthy and glaucomatous eyes in each dataset.

While glaucomatous eyes can range from very mild visual loss to blindness, healthy eyes have a narrower range of possible measurements. Our finding may therefore be an artifact of the restricted-range problem,[29] in which correlations decrease with restricted ranges. To quantify the effect of the restricted-range problem, we performed Pearson correlation on a subset of the DIGS/ADAGES dataset in which we matched the measurement range across groups. We used the DIGS/ADAGES dataset to perform this analysis because it is large enough to allow range restriction while maintaining reasonable levels of statistical power overall (see Table 2). The ranges were matched by generating intervals for the 95% central distribution for MS, MD, RA, and RNFLT for the healthy eyes. We then centered this range onto the median value obtained for the glaucomatous eyes and excluded all values that fell outside that range. In this way, we restricted the range in both healthy and glaucomatous groups of eyes, so that the range in structure and function in any pair was equal to or smaller in the glaucomatous group than in the healthy group. Fig 3 shows the Pearson correlation after matching the ranges. We obtained similar results as those shown in Fig 1, with significant correlations in glaucomatous eyes and no correlations in healthy eyes (Fig 3). Table 2 shows the sample size and statistical power for this analysis. Differences in Pearson correlations cannot be explained by the smaller range of healthy eyes with respect to glaucomatous eyes. It is more likely that the results are due to differences in the SF associations between healthy and glaucomatous eyes.

**Table 2:** Number of observations and power for analysis for the critical value of 0.2 of Pearson correlation after restricting the range for glaucomatous eyes. Since range restriction yields different number of eyes and subjects in each sector, the values reported here are the minimum sample sizes and power over all sectors.

	Healthy eyes		Glaucomatous eyes	
	Number of eyes	Power	Number of eyes	Power
RA vs SAP MS	623	1.00	212	0.84
RA vs SAP MD	623	1.00	201	0.82
RA vs SWAP MS	426	0.99	197	0.81
RA vs SWAP MD	428	0.99	189	0.79
RA vs FDP MS	616	1.00	213	0.84
RNFLT vs SAP MS and MD	181	0.77	129	0.63
RNFLT vs SAP MD	180	0.77	127	0.62
RNFLT vs FDP MS	161	0.72	118	0.59

Although it is commonly assumed that structural defects precede functional defects in glaucoma,[1] large population-based studies have reported that functional defects can be detected before structural defects.[30, 31] There is therefore no consensus that our decision to use structure as the explanatory variable was justified for the linear regression analyses. Furthermore, GEE, LMM, and OLS univariate models assume that the explanatory variable can be not only controlled, but measured without error, two assumptions that are not met by our data. Thus, even though the methods are appropriate to test for an association between structure and function, [32] they lead to biased slope estimates. If we swapped the explanatory variable (structure) and the response variable (function), the fitted line would be different.[21] We therefore performed two additional analyses.

First, we reran the GEE and OLS analyses using function as the explanatory variable. We obtained similar results (supporting information in S3 Fig): stronger associations were systematically observed in glaucomatous but not in healthy eyes. This suggests that our finding is likely not due to our choice of an explanatory variable.

Second, to directly assess the limitation of a lack of an error-free, independent explanatory variable, we used an error-in-variables model. The standardized major axis (SMA), which assumes equal signal-to-noise ratios for both variables, was therefore applied after randomly selecting one eye per subject when both eyes were included in the study. SMA accounts for the measurement errors in both structure and function.[32] *P*-values were calculated to test for differences between SMA slopes for healthy and glaucomatous eyes.[33] We found that the estimated slope for healthy eyes were significantly different from the estimated slope for glaucomatous eyes after Bonferroni correction in 50 out of 62 (81%) of all SF pairs globally and in each sector. This is consistent with the results we obtained using Pearson, Spearman, and Kendall correlations, as well as the GEE, LMM and OLS fits that all showed that the SF relationship is different in healthy and glaucomatous eyes.

In healthy subjects, we systematically found no association between structure and function in both age-corrected (MD) and non-age-corrected (MS) indices. Yet, non-pathologic, age-related loss of retinal ganglion cells occurs.[34–37] This loss has been associated with a decrease in RNFLT of about 2.5 *μ*m per decade [38, 39] and a decrease in RA of about 0.09 mm^2^ per decade,[39] and with a decrease of visual sensitivity of about 0.7dB per decade.[40] The structural parameters included in this study were not corrected for aging effects. We therefore expected some correlation between structure and the non-age-corrected MS index in healthy subjects. Yet, we did not find any significant correlation after Bonferroni correction because the age-related losses are very small compared with inter-individual differences and test-retest variability. After removing the effects of age, i.e. with the MD index, correlations for healthy eyes should be smaller. The Pearson correlation showed this trend, with correlations between structure and MS being slightly greater by about 0.03 in general for all sectors than those observed between structure and MD. Likewise, slopes for the GEE and OLS linear regression models that removed the effect of age by including it as a covariate are smaller than without correction by 0.6% of mean normal function per % of mean normal structure (see supporting information in S5 Fig).

In summary, across all sectors, stronger associations were consistently found in glaucomatous eyes than in healthy eyes using three different datasets, different structure and function pairs, and different statistical approaches. The associations between structure and function are different for healthy and glaucomatous eyes, suggesting that data from these two groups should not be pooled together when assessing the structure-function relationship in glaucoma. Doing so would result in weaker structure-function associations in glaucoma patients. Similarly, approaches using structure and function to model glaucoma progression should consider these differences in associations between structure and function between healthy and glaucomatous eyes.

## Supporting information

S1 Fig Spearman correlation for each of the structure-function pairs. Results are presented globally and in all sectors for healthy (triangles) and glaucomatous (circles) eyes. Correlations that were found to be significantly different from zero after Bonferroni correction are shown in red. Note that the range of the x-axes are different for the different datasets; we plotted the graph using the range observed in each dataset to highlight the differences between healthy and glaucomatous eyes in each dataset.
(TIFF)

S2 Fig Kendall correlation for each of the structure-function pairs. Results are presented globally and in all sectors for healthy (triangles) and glaucomatous (circles) eyes. Correlations that were found to be significantly different from zero after Bonferroni correction are shown in red. Note that the range of the x-axes are different for the different datasets; we plotted the graph using the range observed in each dataset to highlight the differences between healthy and glaucomatous eyes in each dataset.
(TIFF)

S3 Fig Unit effect (slopes) estimated with GEE or OLS regression of structure on function for each of the structure-function pairs. Results are presented globally and in all sectors for healthy (triangles) and glaucomatous (circles) eyes. Slopes that were found to be significantly different from zero after Bonferroni correction are shown in red. Note that the range of the x-axes are different for the different datasets; we plotted the graph using the range observed in each dataset to highlight the differences between healthy and glaucomatous eyes in each dataset.
(TIFF)

S4 Fig Unit effect (slopes) estimated with LMM regression for each of the structure-function pairs. Results are presented globally and in all sectors for healthy (triangles) and glaucomatous (circles) eyes. Slopes that were found to be significantly different from zero after Bonferroni correction are shown in red. Note that the range of the x-axes are different for the different datasets; we plotted the graph using the range observed in each dataset to highlight the differences between healthy and glaucomatous eyes in each dataset.
(TIFF)

S5 Fig Unit effect (slopes) estimated with GEE and OLS regression including age as a covariate for each of the structure-function pairs. Results are presented globally and in all sectors for healthy (triangles) and glaucomatous (circles) eyes. Slopes that were found to be significantly different from zero after Bonferroni correction are shown in red. Note that the range of the x-axes are different for the different datasets; we plotted the graph using the range observed in each dataset to highlight the differences between healthy and glaucomatous eyes in each dataset.
(TIFF)

S1 Data Dataset from the Diagnostic Innovation in Glaucoma Study (DIGS) and from the African Descent and Glaucoma Evaluation Study (ADAGES) studies. (XLSX)

S2 Data Dataset from the IOWA study. (XLSX)

S3 Data Dataset from the SUNY_IU study. (XLSX)
