# Supplementary figures and images for: Associations between structure and function are different in healthy and glaucomatous eyes
Source: PLoS One. 2018 May 3;13(5):e0196814. doi: 10.1371/journal.pone.0196814 (PMC5933752; doi:10.1371/journal.pone.0196814)

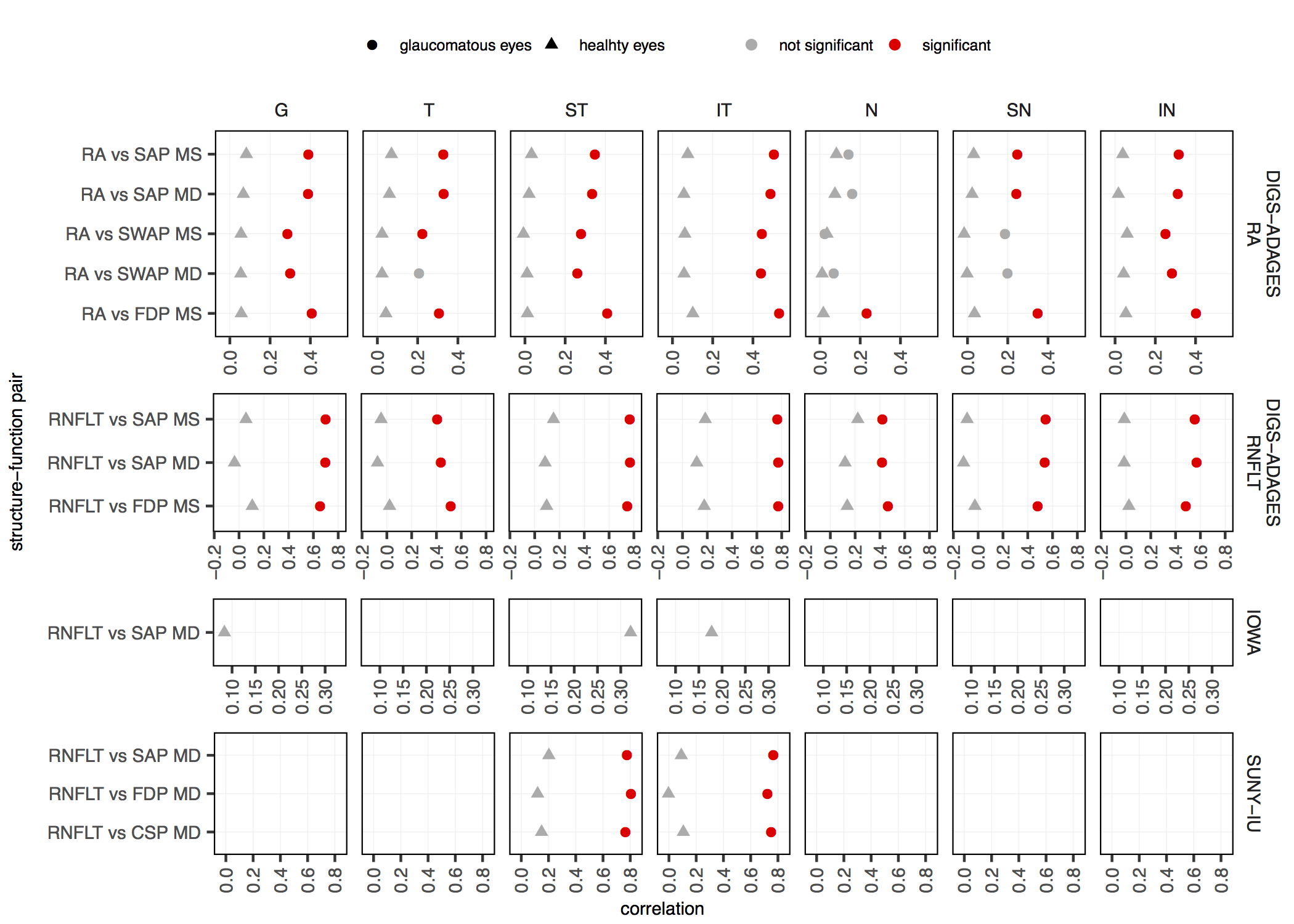

Supplement: S1 Fig — Results are presented globally and in all sectors for healthy (triangles) and glaucomatous (circles) eyes. Correlations that were found to be significantly different from zero after Bonferroni correction are shown in red. Note that the range of the x-axes are different for the different datasets; we plotted the graph using the range observed in each dataset to highlight the differences between healthy and glaucomatous eyes in each dataset. (TIFF) [file pone.0196814.s001.tiff]

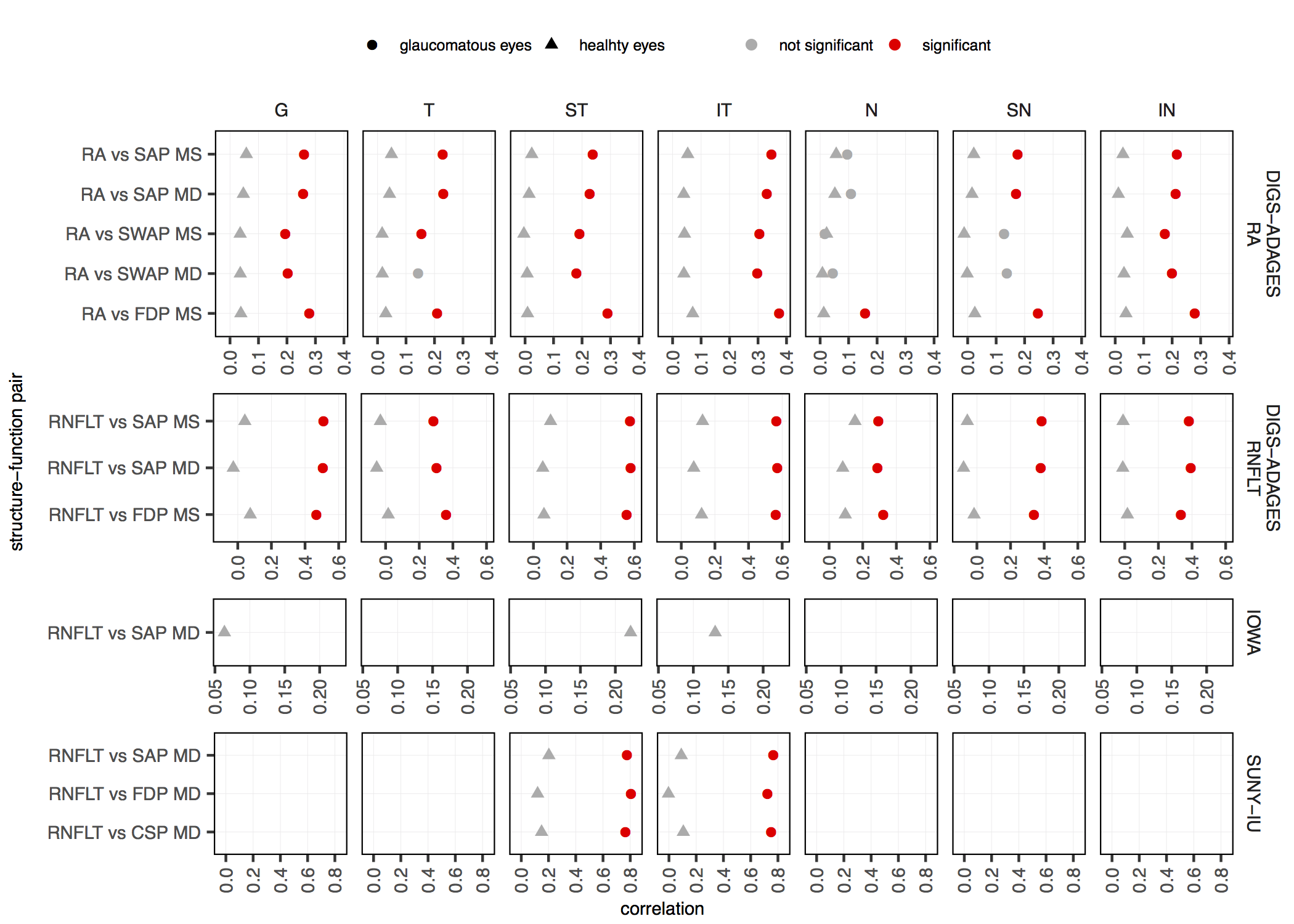

Supplement: S2 Fig — Results are presented globally and in all sectors for healthy (triangles) and glaucomatous (circles) eyes. Correlations that were found to be significantly different from zero after Bonferroni correction are shown in red. Note that the range of the x-axes are different for the different datasets; we plotted the graph using the range observed in each dataset to highlight the differences between healthy and glaucomatous eyes in each dataset. (TIFF) [file pone.0196814.s002.tiff]

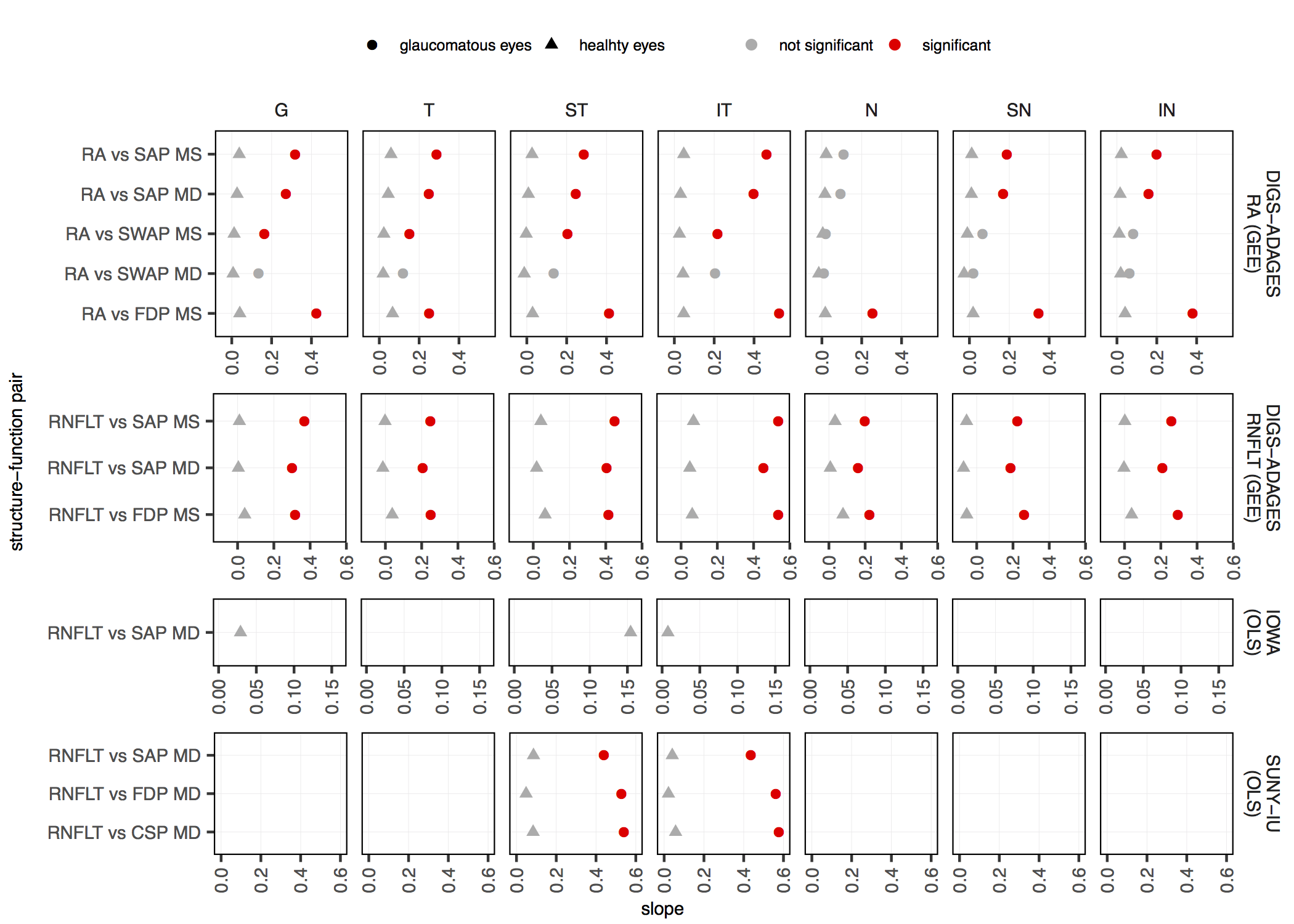

Supplement: S3 Fig — Results are presented globally and in all sectors for healthy (triangles) and glaucomatous (circles) eyes. Slopes that were found to be significantly different from zero after Bonferroni correction are shown in red. Note that the range of the x-axes are different for the different datasets; we plotted the graph using the range observed in each dataset to highlight the differences between healthy and glaucomatous eyes in each dataset. (TIFF) [file pone.0196814.s003.tiff]

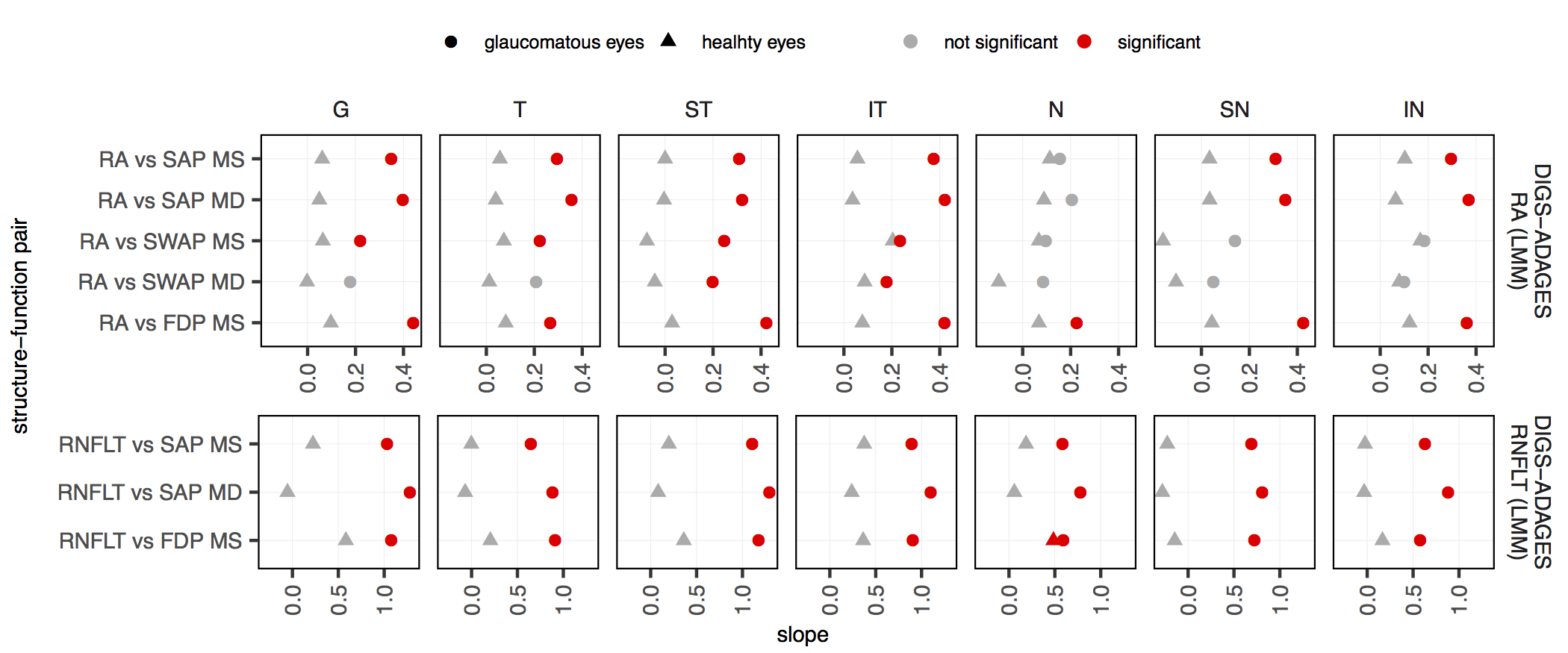

Supplement: S4 Fig — Results are presented globally and in all sectors for healthy (triangles) and glaucomatous (circles) eyes. Slopes that were found to be significantly different from zero after Bonferroni correction are shown in red. Note that the range of the x-axes are different for the different datasets; we plotted the graph using the range observed in each dataset to highlight the differences between healthy and glaucomatous eyes in each dataset. (TIFF) [file pone.0196814.s004.tiff]

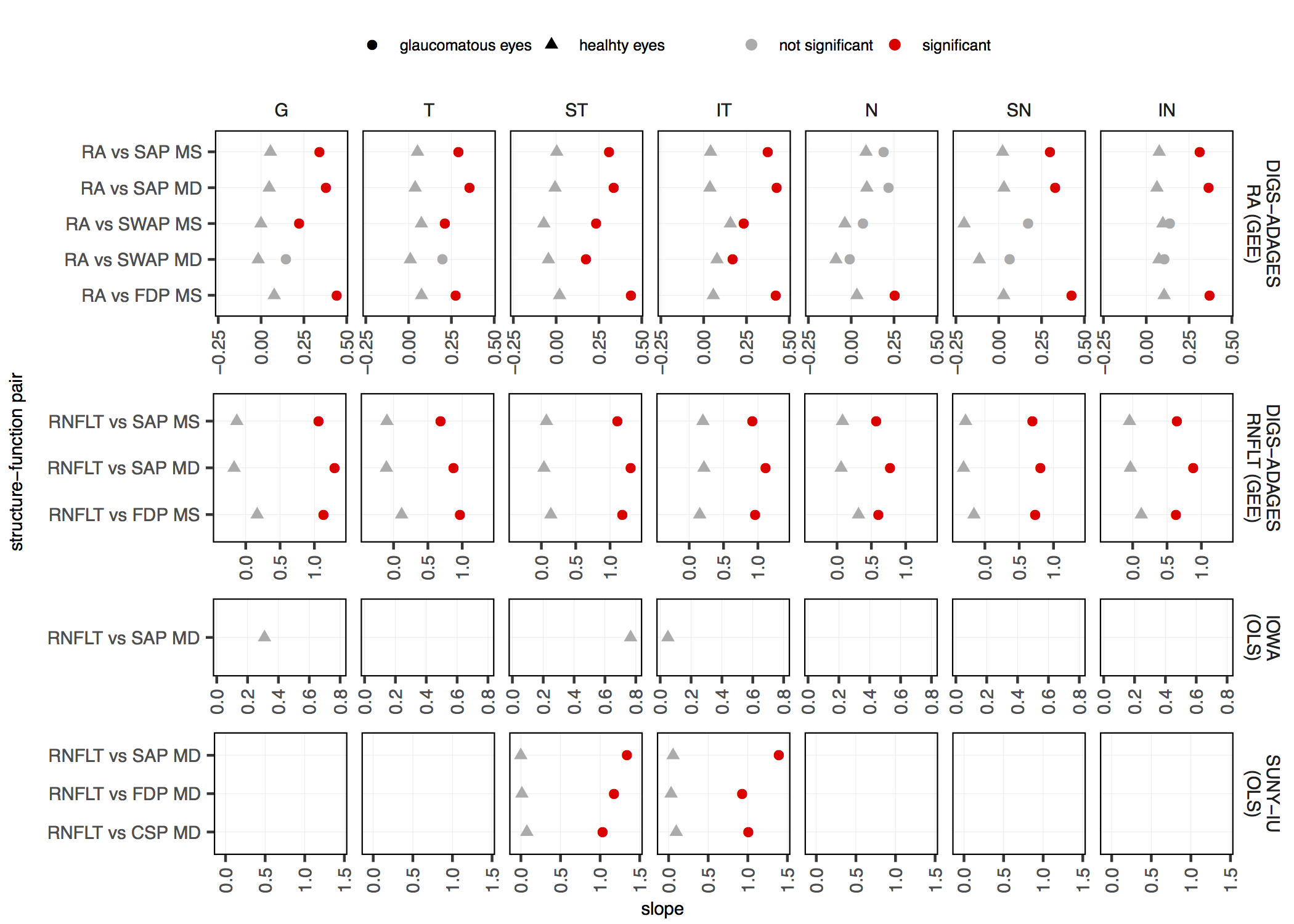

Supplement: S5 Fig — Results are presented globally and in all sectors for healthy (triangles) and glaucomatous (circles) eyes. Slopes that were found to be significantly different from zero after Bonferroni correction are shown in red. Note that the range of the x-axes are different for the different datasets; we plotted the graph using the range observed in each dataset to highlight the differences between healthy and glaucomatous eyes in each dataset. (TIFF) [file pone.0196814.s005.tiff]
